# Delayed Diagnosis of Colonic Adenocarcinoma in a 30-Year-Old Postpartum Woman Misdiagnosed with HELLP Syndrome

**DOI:** 10.1155/2020/3085413

**Published:** 2020-08-27

**Authors:** Alexander J. Maqueira, Ahmad O. Rifai, Courtney Albury, William A. Kantrales, David Rydell, Henry Breland

**Affiliations:** ^1^Alabama College of Osteopathic Medicine, 445 Health Sciences Blvd., Dothan, AL 36303, USA; ^2^The Virtual Nephrologist, Inc., PO Box 1750, Lynn Haven, FL 32444, USA; ^3^Envision Surgical Services, 449 W 23rd St., Panama City, FL 32405, USA; ^4^Emerald Coast OB/GYN, 103 E 23rd St., Panama City, FL 32405, USA

## Abstract

Colon cancer is a rare diagnosis in 30-year-old women, which may be further complicated by their concurrent gravid status. Several physiological changes that occur during an intrauterine pregnancy (IUP) can mask symptoms of early colon cancer. Our patient was a 30-year-old, Gravida 2, Para 0 woman with an uncomplicated pregnancy until the 35^th^ week of gestation when she developed preeclampsia and symptoms suggestive of early hemolysis, elevated liver enzymes, and low platelet (HELLP) syndrome. Following induction of labor and the subsequent, uncomplicated vaginal delivery, the patient developed symptoms of nausea, vomiting, and constipation with abdominal pain and bloating. Abdominal computed tomography (CT) revealed a large mass in the right colon along with the involvement of periaortic lymph nodes and the presence of liver metastases. Hepatic metastases were possibly responsible for the patient's elevated liver enzyme levels, which were initially considered to have been caused by HELLP syndrome because the patient also had preeclampsia. The rarity of colon cancer in young, pregnant patients with no family history, such as in this case, results in poor prognosis owing to nonspecific symptoms of the developing malignancy being attributed to pregnancy, which further delays diagnosis and subsequent therapy. Of 29 cases of colon cancer in pregnant patients recorded till date, this is the first report of a stage 4 adenocarcinoma of the colon with hepatic metastasis, elevated liver enzyme levels, and increased blood pressure with associated preeclampsia, which was diagnosed in the postpartum period. It may be important to consider broader differential diagnoses in expectant patients presenting with unusual and persistent symptoms.

## 1. Introduction

Colon cancer is rare in young adults, and its occurrence in a 30-year-old pregnant patient with no family history prevented its early diagnosis [1]. Colon cancer can cause abdominal pain, nausea, vomiting, and changes in bowel movements, especially if the tumor is large enough to obstruct the intestinal lumen. However, these vague gastrointestinal (GI) symptoms are also associated with a normal intrauterine pregnancy (IUP) [1]. This can delay diagnosis of colon cancer in expectant patients, leading to increased morbidity and mortality, which results in poor prognosis. Colon cancer usually affects patients in their fifties and those older, further lowering the index of suspicion for this diagnosis in a pregnant patient, since child-bearing years usually last only until around the fourth decade of life [2]. Preeclampsia is a common complication occurring in the third trimester of pregnancy, in which an affected patient's blood pressure rises sharply, in association with the presence of both proteinuria and an increased risk of seizures [3]. In this report, we describe the rare case of a colon cancer patient with no known risk factors or family history, who was erroneously suspected to have hemolysis, elevated liver enzymes, and low platelet (HELLP) syndrome due to occurrence of both preeclampsia and elevated liver enzymes, during the third trimester of her pregnancy.

## 2. Case Presentation

A pregnant, 30-year-old G2P0L0 Caucasian woman presented to her obstetrician at 35 weeks and 3 days of gestation with elevated blood pressure in the range of 180s/100 and was referred to labor and delivery triage. Upon admission, a preeclampsia workup was initiated, during which her blood pressure reading was 182/92. A complete blood count (CBC) and urinalysis (to assess the presence of microalbumin and creatinine) were performed. Her protein/creatinine ratio was 1.12. A complete metabolic panel (CMP) report revealed elevated liver enzymes including aspartate transaminase (AST) and alanine transaminase (ALT) at levels of 155(normal range, 15-37) U/L and 84 (normal range, 12-78) U/L, respectively. The CBC report showed a hemoglobin level of 11.7 (normal range, 11.7-15.5) gm/dL with hematocrit of 35.3 (normal range, 33.0-45.0) %. The total bilirubin level was 0.70 (normal range, 0.0-1.0) mg/dL, and the urinalysis was negative for the presence of any bilirubin or red blood cells (RBCs). Considering her clinical presentation and laboratory findings, the patient was diagnosed with preeclampsia. Furthermore, while the patient's platelet count was not reduced (281 (normal range, 130-400) K/mm^3^), the possibility of developing HELLP syndrome or its incomplete presentation was suspected owing to her elevated liver enzyme levels. The patient was induced and experienced an uncomplicated and short labor followed by a normal, vaginal delivery. The neonate was monitored in the neonatal intensive care unit (NICU) for 24 hours, which were uneventful.

**Figure 1:**
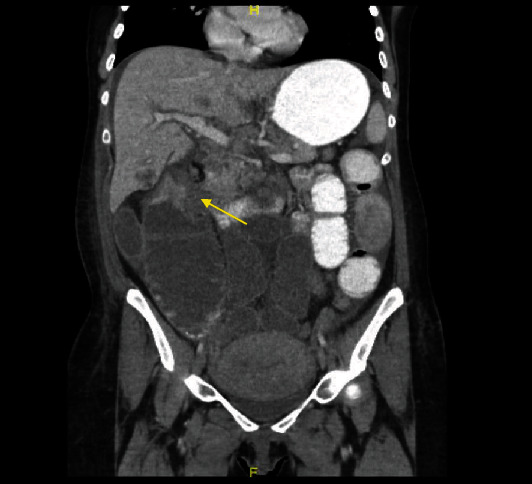
Computed tomography scan showing a mass in hepatic flexure of the colon.

**Figure 2:**
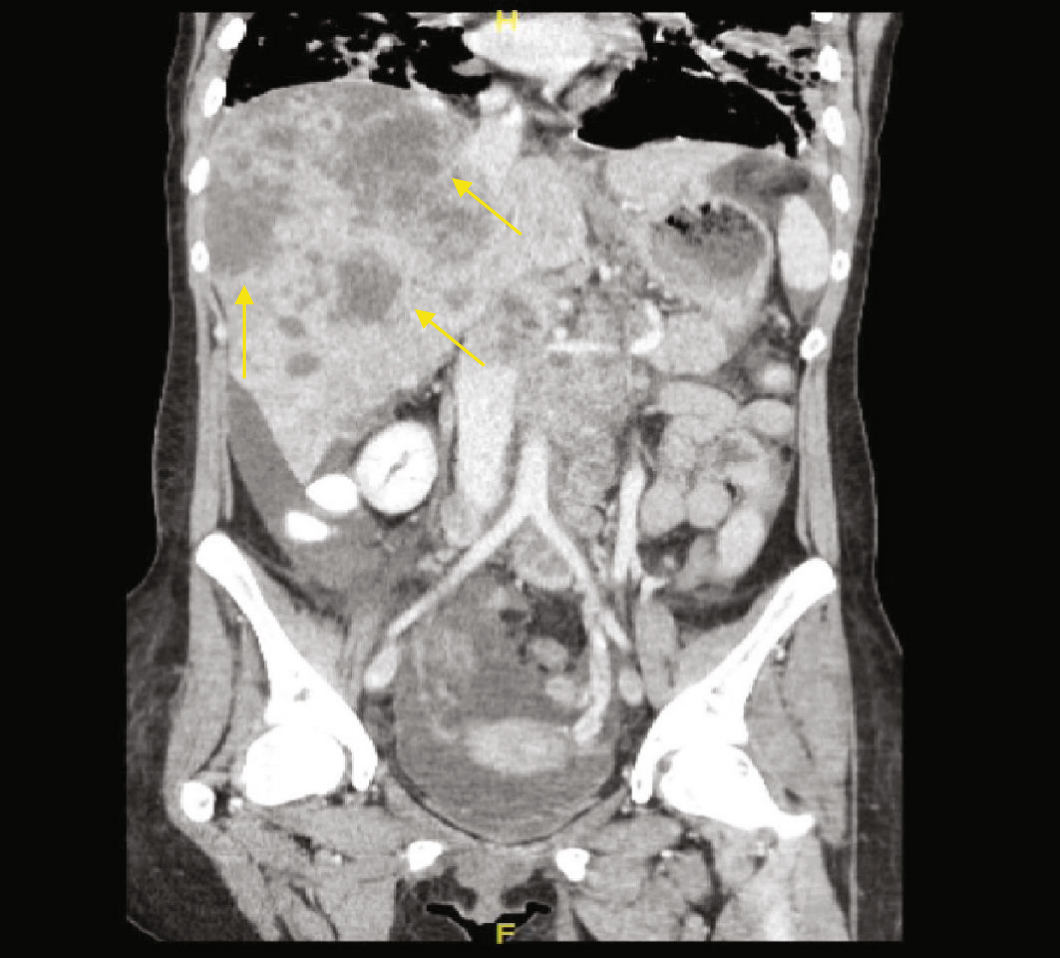
Computed tomography scan showing liver metastasis along with retroperitoneal, mesenteric, and celiac lymphadenopathy.

During the <72-hour postpartum period, the patient began to complain of abdominal discomfort, bloating, nausea, and constipation. On postpartum day 1, the patient was started on magnesium sulfate infusion for 24 hours for seizure prophylaxis and was also started on labetalol, which was continued throughout the hospital stay, for a presumptive preeclampsia. CBC and CMP were repeated, which showed a hemoglobin level of 8.7 gm/dL and a hematocrit of 25.9%. The platelet count increased to 389K/mm^3^, and her AST and ALT levels were 66 U/L and 41 U/L, respectively. These symptoms and lab values are atypical for a postpartum patient, even if they had been diagnosed with preeclampsia or HELLP syndrome, and with the lack of improvement following delivery along with a rising platelet count, it was decided that further investigation was necessary. On postpartum day 6, the patient was obstipated and had not had a bowel movement in 12 days, which prompted an abdominal radiograph. This showed dilated loops of small and large bowel. General surgery was then consulted, and further imaging was ordered. Abdominal and chest computed tomography (CT) revealed a large, 5.6 × 4.1 cm sized mass in the hepatic flexure of the right colon (Figure 1). Additionally, we detected multiple hepatic lesions that were consistent with a finding of liver metastasis, along with the enlargement of retroperitoneal, mesenteric, and celiac lymph nodes. The scans also demonstrated ileus, bilateral pleural effusions, ascites, and involvement of the right hilar, retrocrural, and left supraclavicular lymph nodes (Figure 2). Therefore, a colonoscopy was performed, and biopsy specimens were obtained. Histopathological examination ultimately revealed the diagnosis as poorly differentiated adenocarcinoma of the colon. Pulmonology was consulted for the pleural effusion and recommended giving furosemide. If the effusion did not improve following a course of diuretics, a thoracentesis was recommended but was never performed due to improvement.

Due to the size of the tumor and the fact that tumor debulking would delay our patient getting started with chemotherapy, it was decided that a palliative ileostomy would be most appropriate and debulking would be held off until a later date, if at all. With a stage 4 adenocarcinoma, the utmost importance is starting chemotherapy in a timely fashion. The palliative diverting ileostomy was performed on the following day. The patient's postoperative course was uncomplicated, and the ileostomy was functional. The patient was urgently referred to an oncologist and has since begun combination chemotherapy with fluorouracil, leucovorin, irinotecan, and bevacizumab.

The patient's carcinoembryonic antigen (CEA) level, assessed before discharge, was elevated at 266.0 (normal range, ≤3) ng/mL.

The patient provided informed consent for the publication of her medical information in this case report.

## 3. Discussion

In a review of 29 reported cases of colorectal cancer (CRC) in pregnant patients, Heise et al. reported that the primary tumor most commonly involved the sigmoid colon. While two of the reported patients had developed the primary CRC in the hepatic flexure, they were diagnosed in the antepartum period (i.e., at 18 and 24 weeks of gestation) and were therefore able to consult an oncologist and receive early, systematic treatment [2]. The incidence of patients diagnosed with CRC during pregnancy is 1/18,000 to 1/50,000 (0.002-0.005%) [2]. However, the present narrative is unique because it is the first reported case of a patient with metastatic stage 4 CRC involving the hepatic flexure, who presented with elevated liver enzymes and increased blood pressure associated with preeclampsia in the late, third trimester and was diagnosed in the postpartum period [2]. In the other reported cases of CRC in pregnancy, none of them were complicated by preeclampsia with or without severe features nor were there mentions of hypertension and elevated liver function tests postpartum, suspicious of HELLP syndrome masking the symptoms of CRC with liver metastasis.

Diagnosing colon cancer was challenging in a 30-year-old patient with no family history or known risk factors and presenting with vague symptoms, which could be easily confused with GI complaints commonly associated with a normal, intrauterine pregnancy. The incidence of colon cancer is very low in patients in their childbearing years. This can cause such patients' symptoms to be initially ignored, which can result in a definitive diagnosis at a later stage, when the malignancy is usually more advanced [2].

Older patients with colon cancer are at a lower risk of having positive lymph nodes at the time of diagnosis [4]. Conversely, younger patients are at a higher risk of nodal involvement during diagnosis, as it is usually delayed. Healthcare providers are therefore encouraged to further investigate any unusual GI symptoms occurring in a pregnant patient.

Our patient had preeclampsia, which led to what was believed to be early manifestations of the HELLP syndrome. Preeclampsia is a pregnancy-related disorder associated with increased blood pressure and proteinuria, defined as a protein/creatinine ratio of >0.3 [3]. Preventive measures for preeclampsia have not yet been established, although it has been suggested that administration of low-dose aspirin may decrease its incidence [5]. The patient had been diagnosed with preeclampsia, and the concurrent presence of elevated liver enzymes supported a possible diagnosis of HELLP syndrome. Since our patient did not show decreased platelets, it was plausible that the elevation in liver enzyme levels was due to an alternate cause, which should have been discerned via further investigation. Further, despite achieving an uncomplicated outcome of the pregnancy, the case highlights an interesting point regarding erroneous and biased interpretation of abnormal, antenatal CBC and biochemical test findings (e.g., elevated liver enzymes) that may have occurred due to liver metastasis from the CRC and not as a consequence of preeclampsia and incomplete HELLP syndrome.

The incidence of primary colon cancer (46.9 per 100,000 men and 35.6 per 100,000 women, as estimated between 2009 and 2013) has been decreasing at an accelerated rate since the mid-2000s because of the availability of improved methods of detection and removal of premalignant, colonic polyps [6]. This reduction has paralleled a similar decrease in mortality in patients with colon cancer during the same period [6]. Although incidence of CRC remains much higher in patients with predisposing, heritable conditions (e.g., Lynch syndrome and familial adenomatous polyposis) and in those with an affected first-degree relative, those without these risk factors generally have only a 3-7% risk of sporadically developing this malignancy. Beyond these known risk factors, there are several others that can increase risk of development of primary CRC. New mutations involving associated genomes allow for easier development and evolution of premalignant/malignant polyps. African-Americans are at a 20% greater risk than Caucasians for development of sporadic colon cancer. Additional risk factors include consumption of processed and red meats (based on observational studies), alcohol intake, tobacco exposure, obesity, insulin resistance, and male sex (25% increased risk as compared to females) [6–8].

Anemia, abdominal pain, constipation, nausea, and vomiting are all common signs and symptoms that pregnant patients experience throughout their pregnancy. Unfortunately, these are also common signs and symptoms of early colon cancer. A concurrent pregnancy adds to the difficulties of diagnosing CRC, as it imposes limitations with respect to use of diagnostic modalities such as CT scans and X-rays, which are contraindicated, especially during the first trimester, due to their teratogenic potential. While ultrasonography and magnetic resonance imaging (MRI) are feasible alternatives, their specificity is less than that of CT, and MRI may still present a risk to the developing fetus [9]. This further complicates diagnosis of CRC in early stages in affected, pregnant patients and increases its morbidity and mortality. In patients experiencing suspicious and atypical symptoms such as constipation and bloating, it is imperative for the obstetric team to initiate an early and thorough workup and to avoid administering narcotic analgesics, to prevent exacerbating these symptoms [10].

According to the American Cancer Society (ACS), people with an average risk of CRC should be screened starting from 45 years of age, unless they are positive for any of the risk factors enumerated earlier (e.g., African-American descent, family history, heritable syndromes, and inflammatory bowel disease), in which case regular screening should be initiated earlier [11, 12]. Detection of CRC at an early stage greatly decreases risk of mortality [11]. Furthermore, performing screening for CRC and treating the cancer should only be considered in patients with a life expectancy of ≥10 years [11]. If the patient is deemed eligible for screening, there are several approaches a physician can take including invasive techniques (colonoscopy, CT colonography, and sigmoidoscopy) as well as noninvasive tests (fecal immunochemical test, blood testing, and stool deoxyribonucleic acid (DNA) testing) [12]. While colonoscopy has traditionally been the standard investigation performed for colon cancer screening, there are no reports of randomized controlled trials that support its use in patients ≥ 50 years of age [7]. However, none of these investigations have been proven as superior to the rest as a first-line test, and the investigative approach is generally selected at the discretion of the treating physician [12].

Although invasive investigations are considered safe, there is still an inherent risk of complications that may occur due to factors including preceding bowel preparation, sedatives administered for the procedure, or the procedure itself. Colonoscopy requires prior bowel preparation and is associated with the highest rate of complications such as perforation and GI bleeding. However, it provides the benefit of being able to remove suspected, premalignant polyps during screening. The right colon is not evaluated in a sigmoidoscopy, which precludes discovery of possible cancerous tissue at that location. While this procedure also requires prior bowel preparation, unlike colonoscopy, it is associated with fewer complications and may even be performed without sedation. CT colonography is a relatively new modality that necessitates performing colonoscopy subsequently, for the removal or biopsy of any suspicious lesions detected during the former test. It is a challenging modality to use in older adults because it requires adequate insufflation. However, patients are not required to be sedated, and its results, if negative (absence of suspicious colonic lesions), can help reassure patients without exposing them to the risk of an invasive diagnostic procedure [7].

Unlike invasive procedures, noninvasive investigations are considered as safe, initial tests for detection of CRC. The fecal immunochemical test is more specific and sensitive than the fecal occult blood test. Stool DNA testing has the highest sensitivity and improved specificity, as compared to that of other noninvasive procedures. Negative results of noninvasive tests can be used to reassure patients, without subjecting them to any invasive procedures. However, in the event of a positive result (true/false positive), the patient will have to be investigated further using an invasive procedure, usually a colonoscopy [7].

Our patient did not meet the standards of screening criteria for CRC with respect to factors including age, risk factors, and race. Her symptoms were subtle and could have been caused by the concurrent pregnancy. Considering solely HELLP syndrome as responsible for this patient's elevated liver enzymes (which occurred secondary to liver metastasis) may have delayed the diagnosis even further. Atypical and incomplete presentation of suspected HELLP syndrome clued us to the possibility of occurrence of additional, unusual liver pathology, which the CT scan revealed to be the presence of metastasis.

In conclusion, colon cancer in a patient without family history of the condition (3%) and especially during pregnancy (0.002%) is rare [2]. It is also difficult to diagnose because the presenting symptoms such as bloating, abdominal discomfort, ileus, nausea, and vomiting mimic those that occur during the course of a normal pregnancy. A likely diagnosis of preeclampsia with incomplete HELLP syndrome may prevent further investigation required for the detection of an underlying CRC. In retrospect, we realized that our patient did not have HELLP and the elevation of her hepatic enzymes was due to liver metastasis. Further, our patient's platelet levels remained normal, further decreasing the likelihood of her condition being true HELLP syndrome. In such cases, misdiagnosis of the CRC as a complication of pregnancy (e.g., HELLP syndrome) can cause high morbidity and mortality, resulting in poor prognosis of the malignancy. Unusual and subtle symptoms occurring during pregnancy, especially in multiparous women, should be flagged, and additional workup beyond those prescribed by usual pregnancy guidelines should be considered in order to diagnose conditions which may not be related to the patient's gravid status. Since liver function tests are not a routine part of the first or second trimester panel (as per the patient's obstetrician), it is unknown whether or not these levels would have been abnormal in this period and prompted further evaluation. It is possible that an earlier, thorough evaluation could have potentially helped diagnose the CRC at an earlier stage which would have allowed for the surgical removal of the primary tumor and prevented further spread.
